# Evaluation of safety and efficacy of the bone marrow mesenchymal stem cell and gelatin-nano-hydroxyapatite combination in canine femoral defect repair

**DOI:** 10.3389/fvets.2023.1162407

**Published:** 2023-06-21

**Authors:** Zihang Ma, Xiaoying Guo, Jun Zhang, Qifeng Jiang, Wuying Liang, Wenxin Meng, Shuaijiang Chen, Yufan Zhu, Cundong Ye, Kun Jia

**Affiliations:** ^1^College of Veterinary Medicine, South China Agricultural University, Guangzhou, China; ^2^Guangdong Technological Engineering Research Center for Pet, Guangzhou, China; ^3^Guangdong Polytechnic of Science and Trade, Guangzhou, China; ^4^College of Tropical Agriculture and Forestry, Guangdong Agriculture Industry Business Polytechnic, Guangzhou, China

**Keywords:** canine bone marrow mesenchymal stem cells, gelatin, hydroxyapatite, bone defect, co-culture, bone tissue engineering

## Abstract

Femoral shaft fracture is a common bone trauma in dogs. The limitation of mesenchymal stem cells in bone defect applications is that the cell suspension cannot be fixed to the bone defect site. In the study, our objective was to substantiate the combined application of canine bone marrow mesenchymal stem cells (cBMSCs) and gelatin-nano-hydroxyapatite (Gel-nHAP) and evaluate its therapeutic effect on bone defect diseases in dogs. Experiments were performed to evaluate the following: (1) the porosity of Gel-nHAP; (2) the adhesion of cBMSCs to Gel-nHAP; and (3) the effect of Gel-nHAP on cBMSC proliferation. The efficacy and safety of the combination of cBMSC and Gel-nHAP in the repair of femoral shaft defects were evaluated in animal experiments. The results showed that Gel-nHAP supported the attachment of cBMSCs and exhibited good biocompatibility. In the animal bone defect repair experiment, significant cortical bone growth was observed in the Gel-nHAP group at week 8 (*p* < 0.05) and in the cBMSCs-Gel-nHAP group at week 4 (*p* < 0.01). We demonstrated that Gel-nHAP could promote the repair of bone defects, and the effect of cBMSC-Gel-nHAP on the repair of bone defects was profound.

## 1. Introduction

Under current medical background, various synthetic scaffolds have been widely used in the repair and treatment of bone defects. However, the application of biosynthetic scaffolds still needs to be developed and studied in veterinary clinic. The application of simple synthetic scaffolds has a weak osteoinductive formation ability in bone tissue repair (1). However, biological scaffolds can show advantages in osteoinductive formation, bone conduction, degradability, and plasticity (2). Furthermore, the proper pore size in the biological scaffold is conducive to stem cell attachment and provides high-quality biological support for bone tissue (3).

Bone marrow-derived mesenchymal stem cells (BMSCs) are non-hematopoietic multipotent stem cells derived from the mammalian bone marrow matrix that can differentiate into a variety of cells, such as neurons, cardiomyocytes, myocytes, epithelial cells, and islet cells (4). The primary function of mesenchymal stem cells (MSCs) is to repair defective tissue after tissue or organ damage (5). BMSCs have multi-directional differentiation potential and immune regulation ability; therefore, they have broad application potential in the treatment of degenerative diseases and trauma (6). The MSCs used in this study were canine bone marrow-derived mesenchymal stem cells (cBMSCs).

The main component of gelatin-nano-hydroxyapatite (Gel-nHAP) is hydroxyapatite, which is the main mineral component of natural bone. Because it has chemical and physical properties similar to animal bone tissue, it has good biological activity and bone conductivity (7). However, HAP is too brittle and has poor flexibility, which can be solved by combining with gelatin (8, 9). Gelatin is solid at low temperature, and its surface is porous and rough. This feature, combined with its special amino sequence at the molecular level, makes it easy for cells to adhere (10). The combination of gelatin and nano-hydroxyapatite can promote alkaline phosphatase (ALP) activity and osteocalcin expression and exhibits good biocompatibility (11). When used alone, Gel-nHAP is fragile and its histocompatibility is limited to a short time. Therefore, a multifunctional inductive and invasive cell biological agent capable of combining with Gel-nHAP is required to create a new type of scaffold and to increase the feasibility of Gel-nHAP in clinical practice. For example, the combination of Gel-nHAP and stem cells can effectively promote the reconstruction of the osteogenic microenvironment *in vivo* and promote the osteogenic differentiation of stem cells (12, 13). cBMSCs combined with Gel-nHAP have good efficacy in the repair of animal frontal faces (14, 15).

In small animal clinic, high-intensity impact of fracture caused by car accident, jumping off a building, dog bite, etc., can also leads to soft tissue injury, periosteal tear and exfoliation, bone comminution, and bone loss, with various sequalae, such as difficult clinical treatment, a prolonged bone healing period, and even nonunion (16). In addition, fracture repair surgery without strict aseptic manipulation can also lead to a common cause of bone nonunion in dogs. Osteotomy, autogenous bone transplantation, and artificial synthetic bone tissue transplantation are commonly used for the treatment of orthopedic diseases (17). Such methods are easy to cause secondary injury or take a long time to heal (18). Mesenchymal stem cells have shown promising efficacy in the treatment of inflammation, tissue and organ damage (19). However, the mobility of the stem cell suspension after transplantation to the bone defect site does not allow the stem cells to function stably.

The research, development, and use of cBMSCs combined with Gel-nHAP can contribute to the repair of bone defects in small animals, implant therapy, and management of degenerative joint disease in small animals. Stem cell treatment promotes the repair of bone injury, reduces the difficulty of surgery and postoperative recovery, fills the defects of past autologous tissue filling, that is, “to repair trauma by trauma,” solves the problems of infection and immune rejection of allogeneic bone transplantation, and aids healing (14, 20).

## 2. Materials and methods

### 2.1. Source of cBMSCs and Gel-nHAP

The cBMSCs were obtained from our laboratory (Clinical Surgical Laboratory, College of Veterinary Medicine, South China Agricultural University, Guangzhou, Guangdong, China). Bone marrow blood from the canine femur was collected using methods described earlier (21), and cBMSCs were isolated and cultured. Gelatin (3% biotechnology grade) was melted at 60°C, mixed with nano-hydroxyapatite, lyophilized, cross-linked with N-hydroxysuccinimide and 1-(3-dimethylaminopropyl)-3-ethylcarbodiimide hydrochloride, and then lyophilized again to form the final product. The above reagents were purchased from Shanghai Macklin Biochemical Co., Ltd.

### 2.2. Detection of the attachment ability of cBMSCs on Gel-nHAP

The cBMCSs were cultured to the third passage and collected for scanning electron microscopy, the cell density was adjusted to 1 × 10^6^ cells/mL, and Gel-nHAP was trimmed into small cuboids with a volume of approximately 5 × 5 × 3 mm^3^. cBMSCs were co-cultured with Gel-nHAP in 24-well plates for 7 days. Gel-nHAP with cBMSC (cBMSC-Gel-nHAP) was placed in glutaraldehyde and fixed overnight at 4°C. The next day, the cBMSC-Gel-nHAP was washed, dehydrated using an ethanol gradient series, dried overnight using a critical drying instrument, and then coated under vacuum. Finally, the attachment of cBMSC was observed under a scanning electron microscope.

Acridine Orange (AO, Jiangsu keygen biotechnology service Co. LTD) staining method was used after the co-cultured cBMSCs-Gel-nHAP scaffolds were gently cleaned with phosphate-buffered saline two to three times according to the manufacturer’s instructions. Approximately 500 μL of AO staining solution (95:5 ratio) was added to the 24-well plate, so the scaffolds were immersed in the staining solution and stained for 30 min at 26°C in the dark. Live cell attachment was subsequently observed under a fluorescence microscope with an excitation filter wavelength of 488 nm and a blocking filter wavelength of 515 nm.

### 2.3. Effect of Gel-nHAP on the proliferation capacity of cBMSCs

Cell counting kit-8 (CCK-8, Shanghai hongye biotechnology Co., LTD) assay was used to determine the effect of Gel-nHAP on cBMSC proliferation. The sterilized Gel-nHAP was soaked in MSC culture medium (1 g:10 mL) for 72 h, centrifuged at 800 × *g* for 5 min, and filtered to obtain the extract. The third passage of cBMSCs was spread into 96-well plates at a density of 1 × 104 cells/mL. After adhesion, the control group (MSC culture medium) and the experimental group (Gel-nHAP extract) were established, and each group had three parallel wells. The CCK-8 assay was performed on days 1, 3, 5, and 7, and the optical density (OD) value at 450 nm was measured using a microplate reader (22).

### 2.4. Animal experiments

Twelve healthy adult male beagle dogs were used to establish the femur defect model, divided into sham operation, blank, Gel-nHAP, and cBMSCs-Gel-nHAP groups, with three dogs in each group. In the sham operation group, the skin and fascia of the hind limbs were cut through the superficial fascia lata incision according to the surgical approach, and the biceps femoris and tensor fascia lata were blunt-stripped along the muscle space to expose and peel the periosteum and peel. The surgical site was sutured after exposing the femoral shaft. In the blank group, using the same surgical approach, the right hind limb femur was subjected to fenestration osteotomy, the cortical bone was cut, generating three defects of approximately 8 × 5 mm in size, and then the periosteum, muscle, and skin tissue were sutured. In the Gel-nHAP group, three bone defect gaps were created according to the method described for the blank group, and then sterilized Gel-nHAP was filled into the gaps, and the periosteum, muscle, and skin tissue were sutured sequentially. In the cBMSCs-Gel-nHAP group, three gaps were created like described earlier in the passage, and then Gel-nHAP attached to cBMSCs was filled into the gaps and tissues were sutured sequentially.

#### 2.4.1. Safety evaluation of cBMSCs-Gel-nHAP therapy in dogs

##### 2.4.1.1. Physical examination

The body temperature, respiration, and heart rate of the experimental dogs were examined on the day of modeling and 6 h, 8 h, 10 h, 12 h, 24 h, 3 days, 5 days, and 7 days after modeling. The gait and wound recovery of the dogs were observed.

##### 2.4.1.2. Routine blood test

Blood (0.5 mL) was collected from the forearm vein of the dogs on the day of modeling and 3, 5, and 7 days after. The blood was quickly transferred into EDTA-Na2 tubes for anticoagulation. White blood cell (WBS), neutrophil (NEUT), lymphocyte (LYMPH), monocyte (MONO), eosinophil (EOS), basophil (BASO), red blood cell (RBC), and platelet (PLT) counts, hemoglobin (HGB) levels, and hematocrit (HCT) were determined.

##### 2.4.1.3. Blood biochemical test

Blood (1 mL) was collected from the forearm vein of the dogs on the day of modeling and 1, 3, and 7 days after. Blood was quickly transferred to a lithium heparin anticoagulant tube and centrifuged at 800 × *g* for 5 min to separate the plasma. Total protein (TP), albumin (ALB), globulin (GLOB), total bilirubin (TBIL), creatine kinase (CK), ALP, alanine aminotransferase (ALT), aspartate aminotransferase (AST), glutamyl transpeptidase (GGT), blood urea nitrogen (BUN), creatinine (CRE), and calcium (Ca) levels were measured.

#### 2.4.2. Clinical efficacy of cBMSCs-Gel-nHAP in dogs

##### 2.4.2.1. Evaluation of X-ray imaging

X-rays were taken on the day of modeling and 1 day and 1, 2, 4, and 8 weeks after modeling, and bone healing was analyzed.

##### 2.4.2.2. Semi-quantitative assessment of anatomy and bone formation

At 2, 4, and 8 weeks after modeling, the first, second, and third sites of the modeling were anatomically observed, photographed, and semi-quantitative assessment of bone formation was performed (23) (Table 1).

**Table 1:** Semi-quantitative assessment of bone formation.

Score	Extent of bone present within the transplant
0	No bone evident
1	Minimal bone evident (one trabecula)
2	Weak bone formation, occupying only a small portion of the section
3	Moderate bone formation, occupying a significant portion but less than one half of the section
4	Abundant bone formation, occupying greater than one half of the section

##### 2.4.2.3. Histopathological evaluation

Tissue sampling was performed with the dog under anesthesia. At 2, 4, and 8 weeks after modeling, new bone tissue samples of approximately 8 × 5 mm were cut and fixed in 4% paraformaldehyde. The slices were decalcified, embedded in paraffin, and stained with HE (Thermo Fisher Scientific, Waltham, MA, United States). The degree of new bone formation was observed and analyzed.

### 2.5. Data analysis

The samples among multiple groups were compared using Tukey’s test in a single factor analysis. The experimental results are expressed as the mean ± standard error. *P* < 0.05 was considered statistically significant and *p* < 0.01 was considered extremely significant.

## 3. Results

### 3.1. Gross observation, scanning electron microscopy, and porosity detection of Gel-nHAP

**Figure 1:**
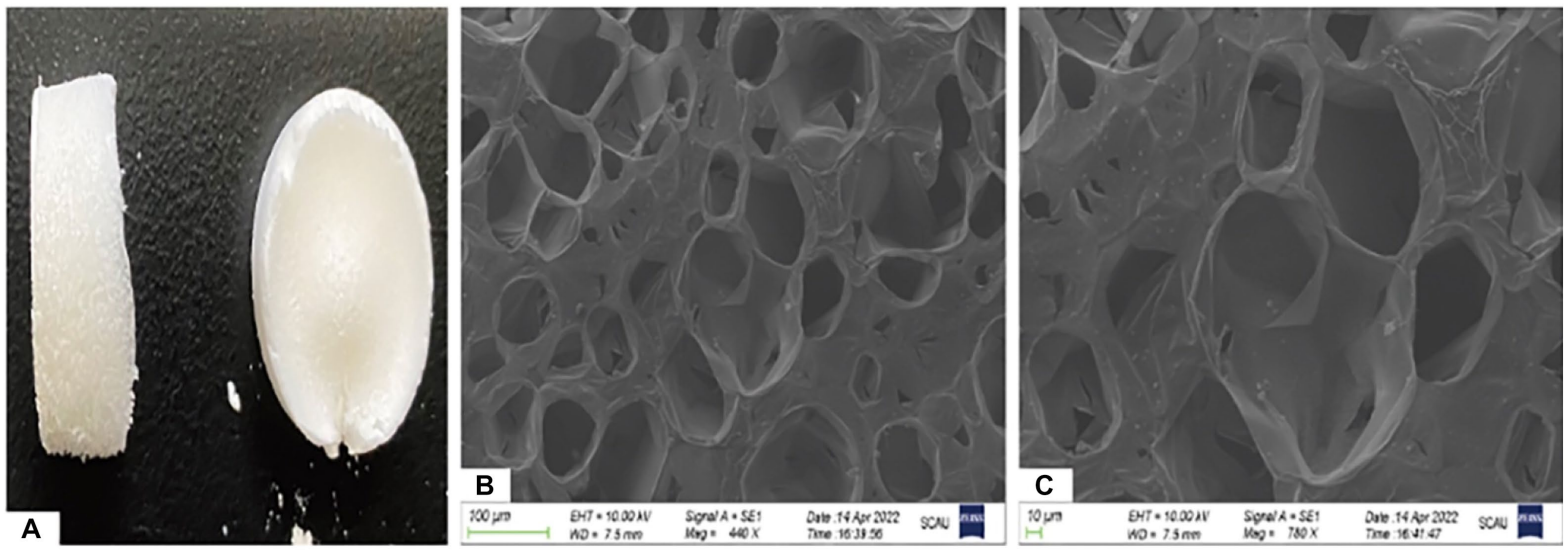
Ocular view and electron microscopic observation of Gel-nHAP. **(A)** Gross observation of Gel-nHAP. **(B)** Gel-nHAP pores under an electron microscope (440×). **(C)** Gel-nHAP pores under an electron microscope (780×).

Gel-nHAP is a milky white spongy solid material. Two samples of scaffold materials were selected and observed under a scanning electron microscope at 440× and 780× magnifications. A porous structure was observed. The pore diameter of the scaffold material was measured using ImageJ software, and the average pore diameter was calculated to be 105.88 ± 11.89 μm (Figures 1A–C). After three parallel experiments, the average porosity of this scaffold batch was 59.27%.

### 3.2. Attachment test of cBMSCs on Gel-nHAP

**Figure 2:**
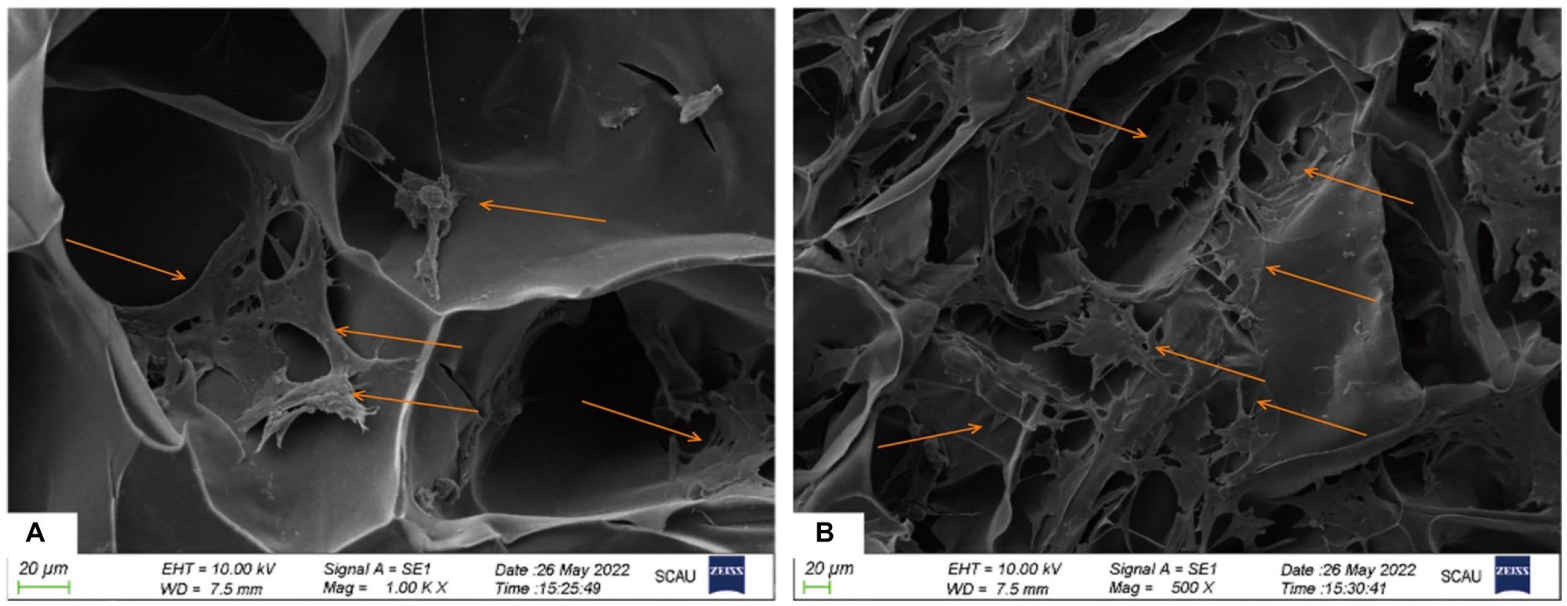
The attachment of cBMSCs to Gel-nHAP under scanning electron microscopy at day 7. **(A)** Numerous cells attached to Gel-nHAP (500×). **(B)** Cells stretched and well attached to Gel-nHAP (1000×).

**Figure 3:**
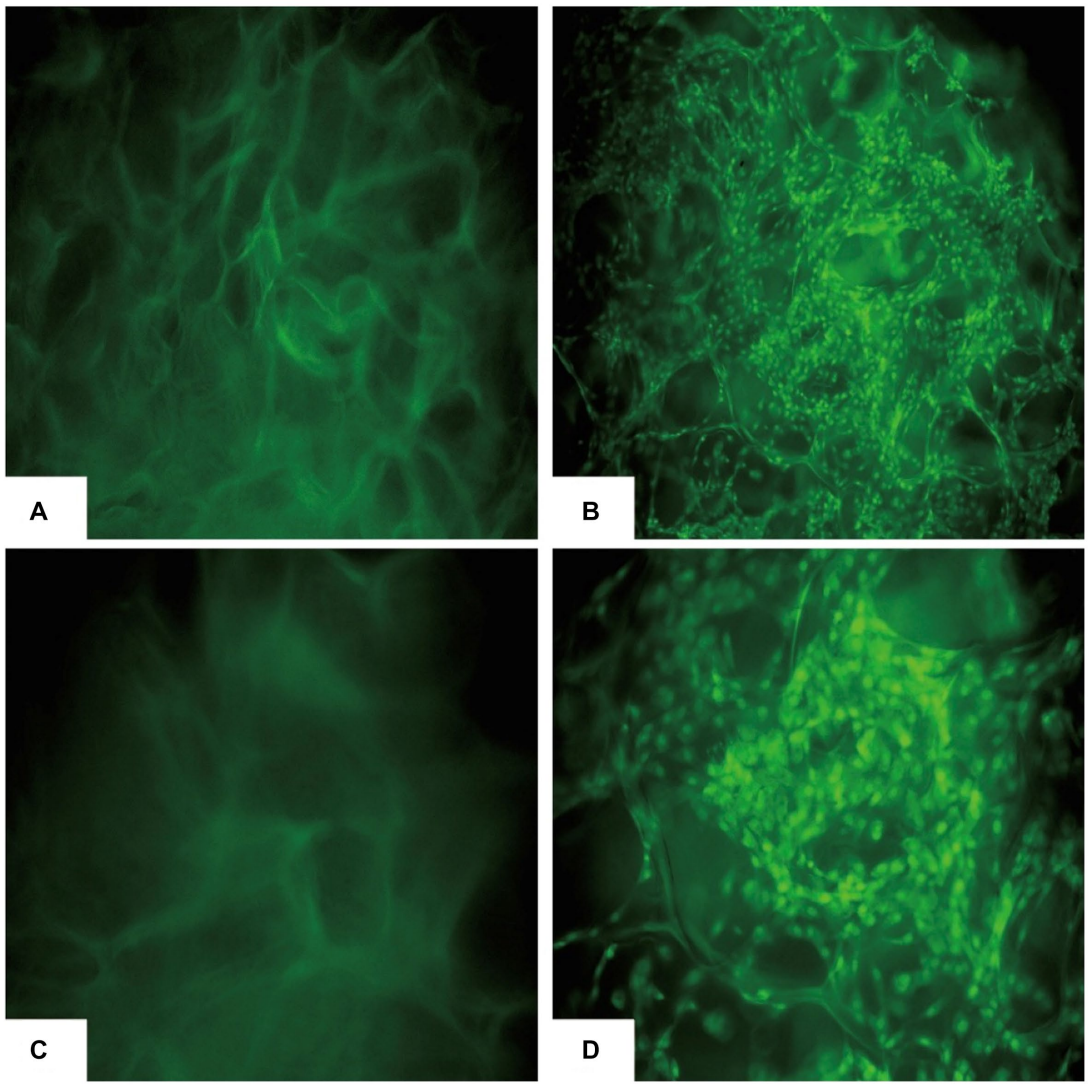
cBMSCs-Gel-nHAP after AO staining. **(A)** Gel-nHAP (50×). **(B)** cBMSCs-Gel-nHAP (50×) cells with dense green fluorescence are viable cBMSCs attached to the scaffold. **(C)** Gel-nHAP (100×). **(D)** Cells with dense green fluorescence in cBMSCs-Gel-nHAP (100×) are viable cBMSCs attached to the scaffold.

The co-cultured samples of cBMSCs and Gel-nHAP for 7 days were observed under scanning electron microscopy at 500× and 1,000× magnification, and numerous cells were observed attached to the scaffold surface with good extension adhesion (Figures 2A,B). After AO staining and compared with Gel-nHAP alone, microscopic observation of cBMSCs-Gel-nHAP showed numerous viable cBMSCs attached to Gel-nHAP (Figure 3).

### 3.3. Effect of Gel-nHAP on cBMSCs proliferation

**Figure 4:**
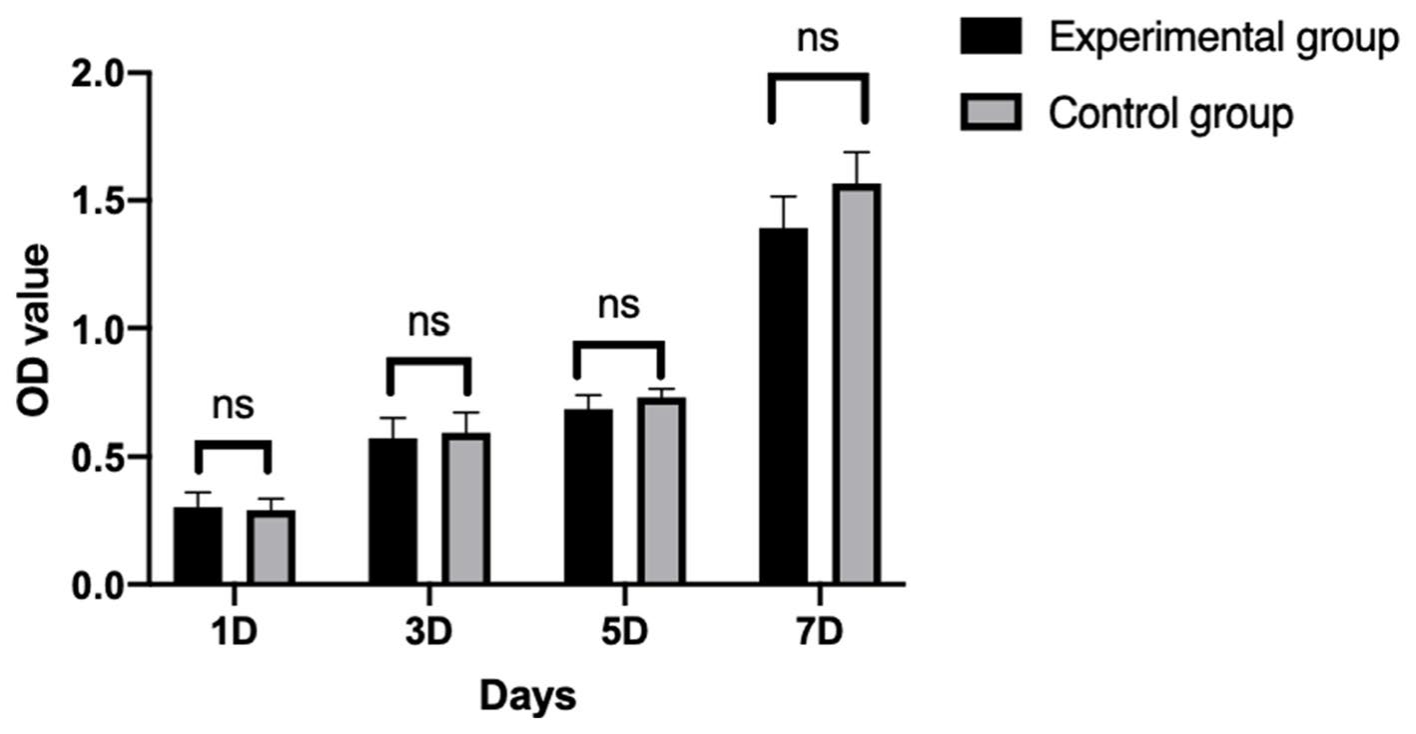
The proliferation ability of cBMSCs (mean ± SE).

After cBMSCs were indirectly co-cultured with Gel-nHAP for 7 days, there was no significant difference in the OD value of the experimental group and the control group on day 1, 3, 5, and 7 as determined by using the CCK-8 assay (*p* > 0.05; Figure 4), and the survival rates of cBMSCs were 100, 94.47, 93.27, and 82.7%, respectively.

### 3.4. Animal clinical trials

#### 3.4.1. Results of the physical examination

Within 7 days of modeling, the test dogs did not show high fever. Within 6–24 h after modeling, the respiratory and heart rates of the dogs in each group increased significantly, and there was no significant difference in body temperature, respiration, and heart rate among the groups (*p* > 0.05). Gait limp appeared within 3 days after modeling in each group; the gait of the sham operation group returned to normal after 3 days and the gait of the other three groups returned to normal after 7 days. After modeling, the wound was swollen, and this symptom lasted for 3–7 days. On day 7, the skin healed well, indicative of primary healing (Supplementary Table S1).

#### 3.4.2. Results of the blood routine test

The WBS and NEUT counts in each group increased significantly on day 1 after modeling and returned to normal after 3 days. The MONO, EOS, BASO, RBC, HGB, HCT, and PLT counts did not show significant changes within 7 days of modeling (Supplementary Table S2). There were no significant differences in routine blood indices among the three groups (*p* > 0.05).

#### 3.4.3. Results of blood biochemical tests

The CK index in each group increased significantly on day 1 after modeling, decreased on day 3, and returned to normal on day 7. ALT levels in the cBMSCs-Gel-nHAP group increased on days 1 and 3 after modeling and returned to normal on day 7. AST levels in the blank, Gel-nHAP, and cBMSC-Gel-nHAP groups increased on day 1 after surgery, then decreased on day 3, and returned to normal on day 7 after surgery. TP, ALB, GLOB, TBIL, ALP, GGT, BUN, CRE, and Ca levels did not show significant abnormalities within 7 days (Supplementary Table S3). There were no significant differences in blood biochemical indices between the groups (*p* > 0.05).

#### 3.4.4. Results of the X-ray evaluation

**Figure 5:**
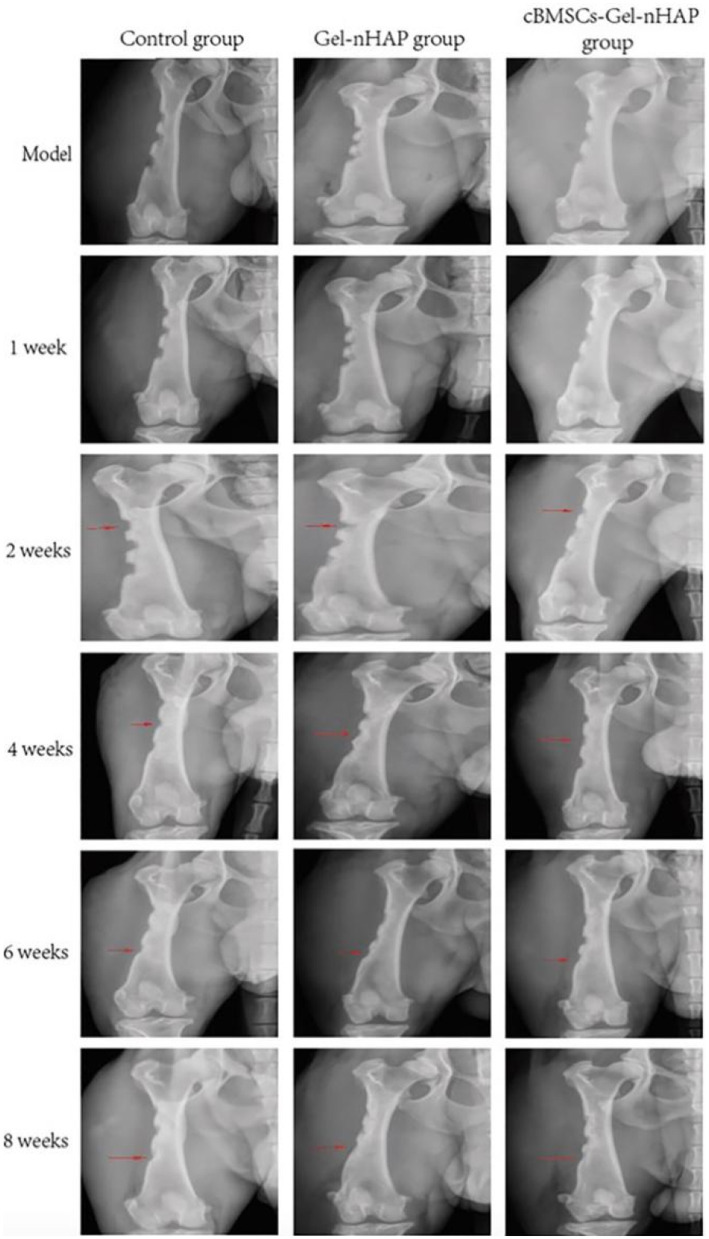
X-ray evaluation results at different periods post modeling. The red arrows indicate the observation sites of bone defect growth at each period, and the arrows also indicate the anatomical observation and sampling sites at 2, 4, and 8 weeks post modeling.

At 1 and 2 weeks after modeling, there was no significant difference in X-ray imaging between the groups. At week 4, there was a clear trend of bone healing in all groups, but there was little difference in the degree of healing between the groups. At weeks 6 and 8, the bone healing of the Gel-nHAP and cBMSCs-Gel-nHAP groups was more obvious than that of the blank group—high-density bone growth was obvious and the bone defect area was reduced. However, the results of the Gel-nHAP group differed significantly from the results of the cBMSC-Gel-nHAP group (Figure 5).

#### 3.4.5. Semi-quantitative assessment of bone formation

**Figure 6:**
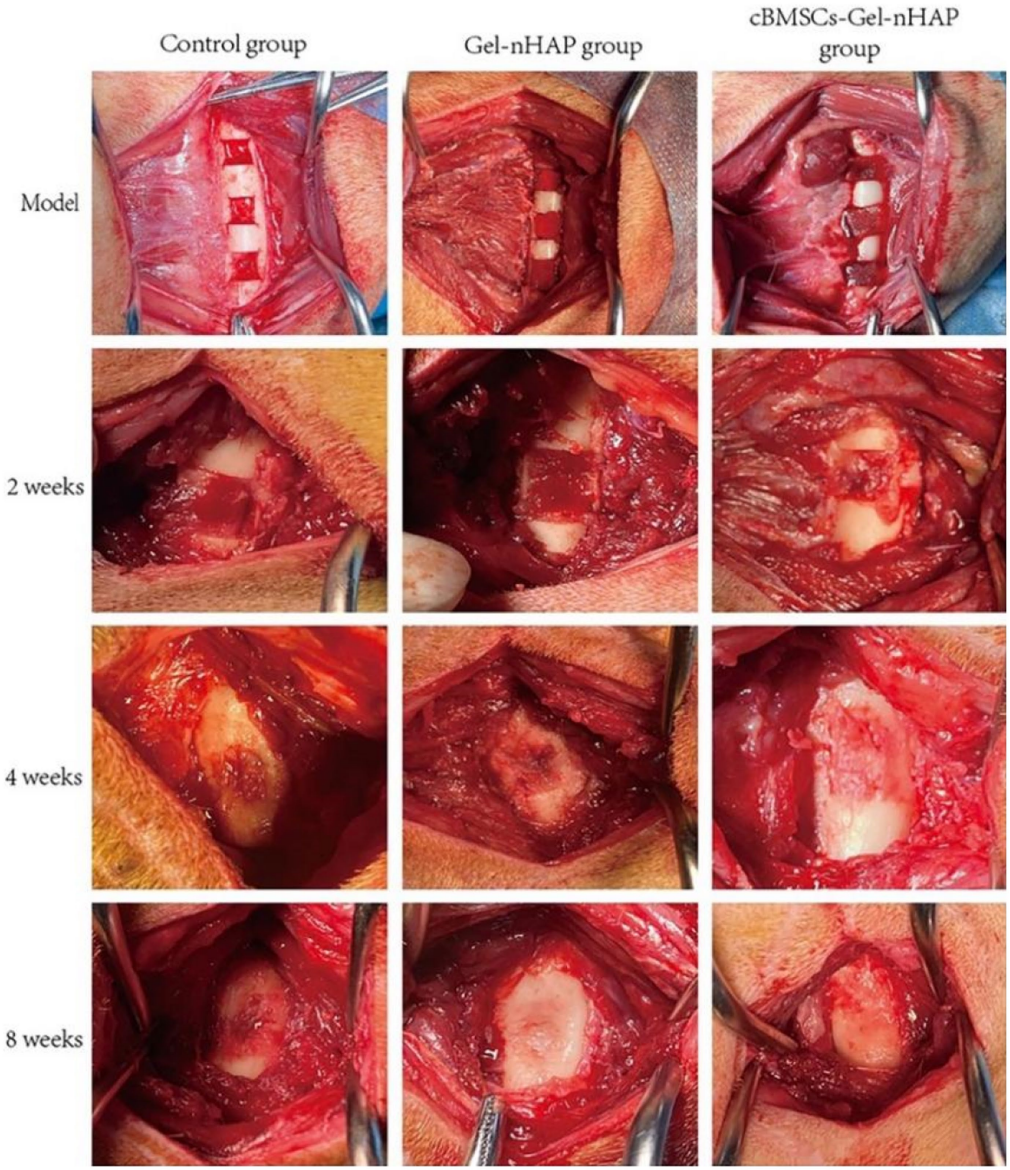
At week 2, there were no significant differences in the bone defect sites of each group. At week, bone tissue had filled the defect in the cBMSCs-Gel-nHAP group. At week 8, bone growth was complete in the Gel-nHAP and cBMSCs-Gel-nHAP groups.

Anatomical observations and sampling were performed on the modeling sites of the experimental dogs at 2, 4, and 8 weeks after modeling (Figure 6). According to the semi-quantitative scoring system of bone formation, there was no obvious bone formation in the blank, Gel-nHAP, and cBMSCs-Gel-nHAP groups at 2 weeks, with a score of 0. At week 4, the blank and Gel-nHAP groups showed weak bone formation, accounting for only a small fraction of the graft area, with a score of 2. In contrast, in the cBMSCs-Gel-nHAP group, bone was formed and occupied the entire graft surface, with a score of 4 (*p* < 0.01). At week 8, bone formation in the blank group represented much, but not more than half, of the grafted part, to a score of 3, compared with the Gel-nHAP and cBMSCs-Gel-nHAP groups, which formed solid bone tissue and occupied the whole grafted surface, with a score of 4 (*p* < 0.05; Table 2).

**Table 2:** Comparison of the results of semi-quantitative scoring of bone formation.

	2 weeks	4 weeks	8 weeks
Control group	0.00 ± 0.00	1.67 ± 0.58	3.00 ± 0.00*
Gel-nHAP group	0.00 ± 0.00	1.67 ± 0.58	4.00 ± 0.00
cBMSCs-Gel-nHAP group	0.00 ± 0.00	4.00 ± 0.00**	4.00 ± 0.00

Significance was measured using the rank-sum test. Values are expressed as mean ± standard error for *n* = 3. Significant differences between the groups at the same time point were analyzed (*p < 0.05, **p < 0.01).

#### 3.4.6. Histopathological evaluation

**Figure 7:**
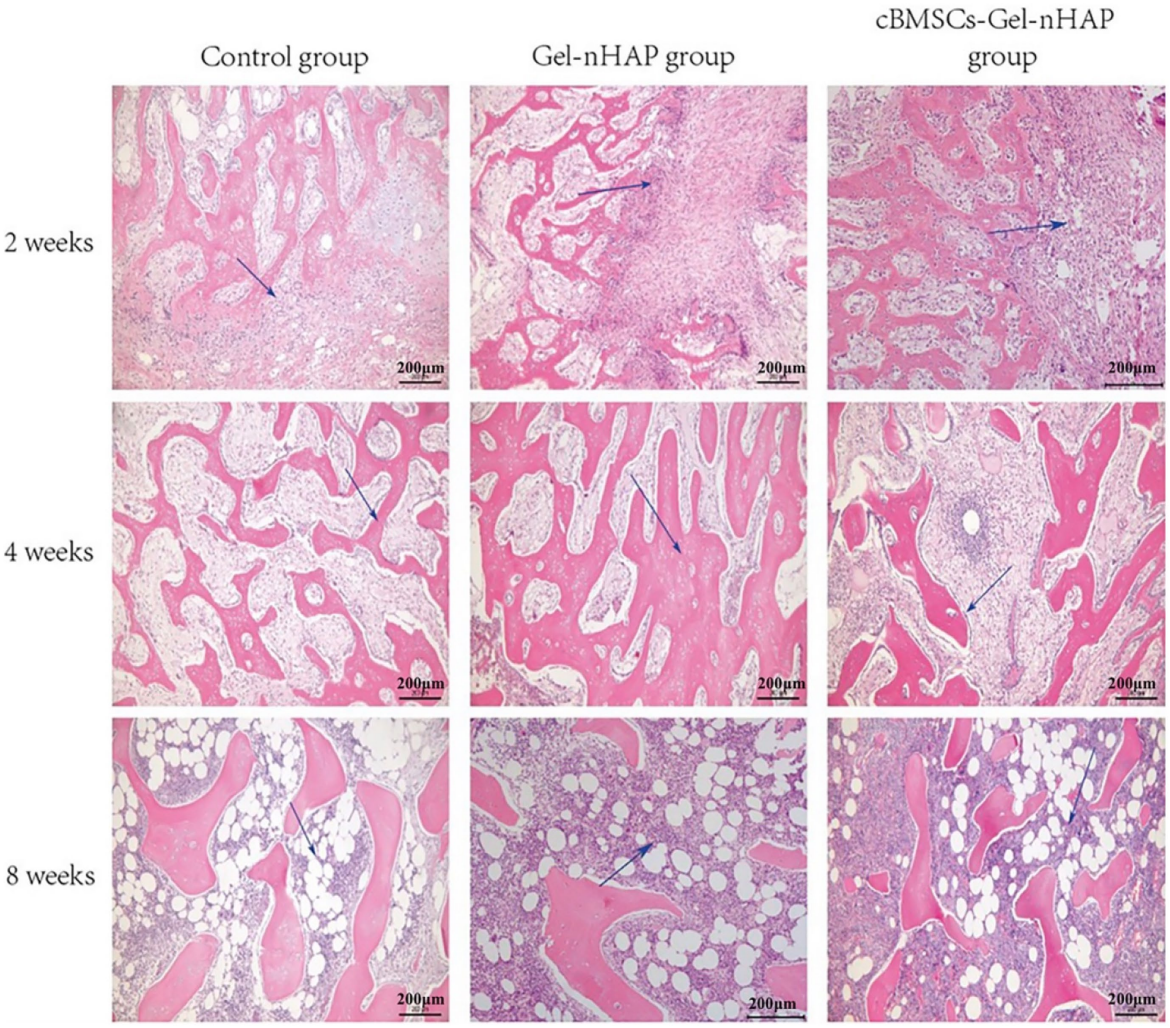
HE-stained bone tissue after defect repair. At 2 weeks, the blue arrow indicates the original connective tissue in the blank group and the new bone tissue in the Gel-nHAP and cBMSC-Gel-nHAP groups. At 4 weeks, the blue arrow indicates the thickening of bone trabeculation in the Gel-nHAP group and the distribution of bone marrow-like tissue in the cBMSC-Gel-nHAP group. The blue arrow indicates the formation of bone marrow cells in each group at week 8.

At week 2, only primitive connective tissue proliferation was observed in the blank group, new bone formation was observed in the Gel-nHAP group, and a large amount of new bone formation was observed in the cBMSC-Gel-nHAP group. At 4 weeks, trabecular bone formed. Compared with the blank group, the trabecular bone in the Gel-nHAP group was significantly thicker, whereas blood vessels and bone marrow tissue were formed and distributed between the trabecular bone in the cBMSCs-Gel-nHAP group. At 8 weeks, the bone marrow cavity was formed and the number of bone marrow cells in the Gel-nHAP group was significantly higher than in the blank group. There was no significant difference between the cBMSCs-Gel-nHAP group and normal bone marrow (Figure 7).

## 4. Discussion

In clinical practice, it is difficult to repair powdered fracture caused by high-energy injury. Usually, delayed bone union or nonunion is caused by severe damage to the blood supply of the bone or incomplete reduction after reduction. To increase the treatment options for bone defect repair, a convenient and low-cost method was sought to optimize the existing bone defect treatment methods, to promote the growth and repair of the femoral defect. Furthermore, the research and application of mesenchymal research cells do not involve ethics, and the immune rejection caused by mesenchymal research cells is relatively small (24, 25). This study verified the effect of a low-cost gelatin/nano-hydroxyapatite scaffold combined with canine BMSCs on the repair of canine femoral defects and confirmed the efficacy of BMSCs combined with gelatin-hydroxyapatite in promoting bone repair.

In bone tissue engineering research, seed cells are the most important research basis. Bone marrow-derived mesenchymal stem cells are the preferred seed cells in bone tissue engineering research due to their convenient materials and abundant sources (26, 27). BMSCs are stem cells derived from mesoderm and are widely distributed in multiple tissues throughout the body. During embryonic development, the mesoderm will develop into the dermis, muscle, bone, and other connective tissues and circulatory system of the body (28). In bone tissue engineering, it has the potential of osteogenic differentiation to accelerate osteogenic repair of bone defects. Furthermore, MSCs have a certain effect on the treatment of inflammation, immune deficiency, and tissue or organ damage (29, 30).

When determining animal models, different scholars have reported different critical values for bone defects. In a study on the repair of skull defects, it was determined that the level of self-healing level of defects with a diameter greater than 8 mm varies between 2 and 12 weeks, which is suitable for biological research on bone scaffolds (31, 32). In this experiment, a rectangular bone defect model was used, which was determined to be 8 mm × 5 mm in size. To ensure that the femur would not fracture, the level of difference in the self-healing of the femur in beagle dogs was determined within 28 weeks, which was in line with the evaluation model after stent transplantation.

Similar to collagen and amino acid composition, gelatin is a macromolecular substance with specific cell recognition sites, which is one reason gelatin is more conducive to cell adhesion than other scaffold materials (33). Relevant literature shows that gelatin, as a common raw material of scaffold materials in tissue engineering, can not only improve the adhesion ability of cells on the scaffold but also improve the viscosity, permeability, and extensibility of scaffold materials, to enhance the cell capacity (10). In addition, the pore size of the scaffold can also determine cell attachment. The required pore size of the scaffold is between 100 and 400 μm, and the number of cell attachments is positively correlated with the pore size (3). Studies have shown that gelatin is used alone to prepare a cell microcarrier with a hollow shell-like structure, which shows good biocompatibility (25). The degradation rate *in vitro* exceeded 70% in approximately 4 weeks (33). Nano-hydroxyapatite (nHAP) is the main mineral component in bone, accounting for 50–70% of the mineral composition of bone tissue (34), and therefore it has good biocompatibility. Compared with conventional HAP with larger particles, nHAP has better biological adaptability, such as adhesion, diffusion ability, and conduction. The combination of gelatin and hydroxyapatite is beneficial to simulate bone components and promote bone repair, so the material application can be well integrated with natural bone components, which can better promote the osteogenic differentiation of MSCs and improve the bone conduction performance of osteoblasts (35). Through direct and indirect co-culture *in vitro*, cBMSCs adhered well to Gel-nHAP. The survival rate of cBMSCs cultured in Gel-nHAP extract was like the growth curve of cBMSCs cultured in normal culture (21), which confirmed that Gel-nHAP had little effect on the proliferation ability of cBMSCs. The compatibility of cBMSCs and Gel-nHAP was good *in vitro*, which met the needs of transplantation.

The process of establishing and repairing a bone defect model causes damage to the dog’s femur and surrounding soft tissue. Postoperative inflammation leads to wound swelling and increased white blood cell count, while pain leads to claudication and increased respiratory rate and heart. The results of this study showed that the total number of white blood cells and neutrophils in the sham-operated group, blank group, Gel-nHAP group and cBMSCs-Gel-nHAP group increased and returned to normal after 3 days, and there was no significant difference among the groups (*p* > 0.05). The establishment of the bone defect model caused damage to the skeletal muscle at the surgical site, leading to an increase in CK index. AST is mainly derived from skeletal muscle, brain, liver, kidney and heart tissue, and skeletal muscle injury is also considered to be the cause of elevated AST activity. Serum ALP activity is generally considered to be liver-specific, but is occasionally increased by muscle injury, and correlation with serum creatine kinase can distinguish between the increase in ALP activity caused by muscle injury and that caused by liver injury (36). The results of this study showed that ALP, AST and CK in Gel-nHAP group and cBMSCs-Gel-nHAP group increased and returned to normal after 3 days, and there were significant differences between the groups (*p* < 0.05). The reason for this result may be that the skeletal muscle was damaged by prolonged traction of the retracter during implantation. X-ray can be used to observe the healing of bone defects. Relevant studies have shown that the formation of new bone tissue can be observed by X-ray after 4–6 weeks of implantation of calcium citrate in rabbit femoral defects, and the observation period is recommended to be at least 8 weeks (37). X-ray scan results showed that the thickness and density of the cortical bone in the Gel-nHAP group and the cBMSCs-Gel-nHAP group increased after 6 weeks. In conclusion, animal experiments confirmed that Gel-nHAP could promote bone defect repair.

The advantages of the cBMSCs-Gel-nHAP scaffold in the repair of canine femoral defects lie mainly in the following aspects: (1) The cBMSCs-Gel-nHAP scaffold is simple to fabricate and easily operated by the surgeon. The surgeon can tailor the scaffold according to the specific shape and size of the bone defect to make the scaffold more suitable for the bone defect. (2) The biocompatibility of cBMSCs-Gel-nHAP was good. No serious adverse reactions, such as infection and immune rejection, occurred in all dogs within 1–8 weeks after transplantation.

In the application of Gel-nHAP, the mechanical support of gelatin is poor, and nHAP increases its fragility. Therefore, Gel-nHAP can be used only as a cell scaffold to provide a physiological environment for cells with repair and anti-inflammatory functions, such as cBMSCs, and cannot be used as a mechanical support scaffold to support bone defects. In clinical practice, it is still necessary to use bone plate fixation combined with AO and BO principles to promote the repair of old fractures or bone defects.

Bone tissue engineering biological scaffolds are moving from single to composite, from macroscopic repair to bystander repair, especially when combined with stem cells, to form accurate, effective, non-toxic, safe, and efficient treatment methods. In addition, the treatment of infectious bone diseases, such as osteomyelitis, is also one indication of biological scaffolds.

## 5. Conclusion

This study has confirmed the effectiveness and safety of Gel-nHAP and cBMSCs-Gel-nHAP in bone repair *in vivo*, which meets the conditions of the microenvironment *in vivo*, and provides a reference for the clinical treatment of infectious bone diseases and bone defects.

## Data availability statement

The original contributions presented in the study are included in the article/Supplementary material, further inquiries can be directed to the corresponding authors.

## Ethics statement

The animal study was reviewed and approved by animal breeding facility owned by the Laboratory Animal Center of the South China Agricultural University (protocol code 2020B108).

## Author contributions

KJ and CY: conceptualization. ZM: methodology, software, writing—original draft preparation, and writing—review and editing. ZM, JZ, WL, WM, SC, YZ, and QJ: validation. XG: formal analysis. WM: investigation. JZ: resources and data curation. WL: visualization. SC: supervision. YZ: project administration. QJ: funding acquisition. All authors have read and agreed to the published version of the manuscript.

## Funding

This research was funded by Guangdong Provincial Key Research Platform and Young Innovative Talents Research Program, grant number 2018GKQNCX148.

## Conflict of interest

The authors declare that the research was conducted in the absence of any commercial or financial relationships that could be construed as a potential conflict of interest.

## Publisher’s note

All claims expressed in this article are solely those of the authors and do not necessarily represent those of their affiliated organizations, or those of the publisher, the editors and the reviewers. Any product that may be evaluated in this article, or claim that may be made by its manufacturer, is not guaranteed or endorsed by the publisher.
