# Supplementary material for: Evaluation of safety and efficacy of the bone marrow mesenchymal stem cell and gelatin-nano-hydroxyapatite combination in canine femoral defect repair
Source: Front Vet Sci. 2023 Jun 21;10:1162407. doi: 10.3389/fvets.2023.1162407 (PMC10320857; doi:10.3389/fvets.2023.1162407)
Supplement: Supplementary file 1 [file Table_1.DOCX]

Supplementary Material

Evaluation of Safety and Efficacy of the Bone Marrow Mesenchymal Stem Cell and Gelatin-Nano-Hydroxyapatite Combination in Canine Femoral Defect Repair

Zihang Ma*, Xiaoying Guo, Jun Zhang, Qifeng Jiang,Wuying Liang, Wenxin Meng, Shuaijiang Chen, Yufan Zhu, Cundong Ye and Kun Jia

*** Correspondence:** Kun Jia: [jiakun@scau.edu.cn](mailto:jiakun@scau.edu.cn); Cundong Ye: [cdye@gdaib.edu.cn](mailto:cdye@gdaib.edu.cn)

# Supplementary Figures and Tables

Table S1 Physical examination（mean±SEM）

| Time | Group | Temperature | Respiratory rate | Heart rate | Gait | Wound healing |
| --- | --- | --- | --- | --- | --- | --- |
| **Before modeling** | **Sham-operated group** | 38.5±0.4 | 34±4 | 122±4 | Normal | / |
|  | **Ccontrol group** | 38.6±0.2 | 36±2 | 103±4 | Normal | / |
|  | **Gel-nHAP group** | 39.0±0.4 | 39±5 | 121±11 | Normal | / |
|  | **cBMSCs-Gel-nHAP group** | 38.7±0.2 | 36±5 | 115±6 | Normal | / |
| 6 h | **Sham-operated group** | 38.2±0.3 | 40±5 | 139±11 | Limp | Swollen |
|  | **Ccontrol group** | 38.5±0.3 | 44±3 | 114±6 | Limp | Swollen |
|  | **Gel-nHAP group** | 38.3±0.3 | 42±5 | 139±11 | Limp | Swollen |
|  | **cBMSCs-Gel-nHAP group** | 39.0±0.4 | 41±6 | 142±3 | Limp | Swollen |
| 8 h | **Sham-operated group** | 39.2±0.5 | 41±4 | 143±8 | Limp | Swollen |
|  | **Ccontrol group** | 38.5±0.4 | 40±7 | 143±11 | Limp | Swollen |
|  | **Gel-nHAP group** | 38.7±0.5 | 43±5 | 139±9 | Limp | Swollen |
|  | **cBMSCs-Gel-nHAP group** | 38.7±0.4 | 46±6 | 140±4 | Limp | Swollen |
| 10 h | **Sham-operated group** | 38.5±0.9 | 44±5 | 137±10 | Limp | Swollen |
|  | **Ccontrol group** | 39.0±0.4 | 42±9 | 139±7 | Limp | Swollen |
|  | **Gel-nHAP group** | 39.0±0.5 | 43±2 | 138±4 | Limp | Swollen |
|  | **cBMSCs-Gel-nHAP group** | 39.0±0.5 | 43±3 | 144±5 | Limp | Swollen |
| 12 h | **Sham-operated group** | 38.7±0.5 | 41±6 | 141±4 | Limp | Swollen |
|  | **Ccontrol group** | 39.2±0.1 | 41±2 | 133±8 | Limp | Swollen |
|  | **Gel-nHAP group** | 39.2±0.2 | 42±5 | 130±16 | Limp | Swollen |
|  | **cBMSCs-Gel-nHAP group** | 39.6±0.1 | 42±1 | 138±2 | Limp | Swollen |
| 24 h | **Sham-operated group** | 39.2±0.6 | 39±8 | 125±13 | Limp | Swollen |
|  | **Ccontrol group** | 39.1±0.2 | 38±6 | 134±12 | Limp | Swollen |
|  | **Gel-nHAP group** | 39.1±0.6 | 36±2 | 121±15 | Limp | Swollen |
|  | **cBMSCs-Gel-nHAP group** | 39.4±0.3 | 42±3 | 118±7 | Limp | Swollen |
| 3 D | **Sham-operated group** | 38.5±0.2 | 39±8 | 117±11 | Normal | Slightly swollen |
|  | **Ccontrol group** | 38.3±0.5 | 37±1 | 102±12 | Limp | Slightly swollen |
|  | **Gel-nHAP group** | 38.9±0.1 | 38±2 | 103±12 | Limp | Slightly swollen |
|  | **cBMSCs-Gel-nHAP group** | 38.5±0.6 | 45±5 | 111±9 | Limp | Slightly swollen |
| 5 D | **Sham-operated group** | 38.2±0.4 | 37±1 | 107±12 | Normal | Well healed |
|  | **Ccontrol group** | 38.5±0.3 | 35±1 | 99±3 | Normal | Well healed |
|  | **Gel-nHAP group** | 38.8±0.3 | 34±3 | 97±8 | Normal | Well healed |
|  | **cBMSCs-Gel-nHAP group** | 38.5±0.2 | 44±4 | 109±4 | Normal | Well healed |
| 7 D | **Sham-operated group** | 38.2±0.4 | 38±9 | 105±3 | Normal | Well healed |
|  | **Ccontrol group** | 38.3±0.2 | 41±4 | 107±4 | Normal | Well healed |
|  | **Gel-nHAP group** | 38.8±0.3 | 38±2 | 115±6 | Normal | Well healed |
|  | **cBMSCs-Gel-nHAP group** | 38.5±0.2 | 41±4 | 114±12 | Normal | Well healed |

Table S2 Blood routine examination results（mean±SEM）

| **Indicators**  **（Unit）** | **Group** | **Before modeling** | **1D** | **3D** | **5D** | **7D** | **Normal range** |
| --- | --- | --- | --- | --- | --- | --- | --- |
| WBC（10^9^/L） | **Sham-operated group** | **9.40±1.98** | **19.15±1.06** | **11.29±1.05** | **12.56±1.37** | **11.14±0.35** | 5～17 |
|  | **Ccontrol group** | **10.08±1.68** | **18.94±2.93** | **11.42±0.86** | **13.53±2.14** | **9.94±2.56** |  |
|  | **Gel-nHAP group** | **8.72±0.91** | **17.85±2.04** | **13.93±1.78** | **12.81±0.87** | **11.2±1.07** |  |
|  | **cBMSCs-Gel-nHAP group** | **10.12±2.52** | **13.60±2.61** | **13.60±2.61** | **16.42±4.12** | **10.86±1.89** |  |
| NEUT（10^9^/L） | **Sham-operated group** | **6.13±1.62** | **15.55±0.92** | **7.49±0.48** | **7.10±0.21** | **6.33±1.43** | 2.7～12.3 |
|  | **Ccontrol group** | **6.67±1.51** | **15.82±2.28** | **7.96±0.21** | **10.24±4.90** | **6.97±2.35** |  |
|  | **Gel-nHAP group** | **5.77±1.25** | **14.66±1.96** | **9.91±1.58** | **9.37±0.70** | **7.97±0.94** |  |
|  | **cBMSCs-Gel-nHAP group** | **6.22±2.05** | **18.37±2.70** | **9.80±2.05** | **11.82±3.24** | **7.31±1.79** |  |
| LYMPH（10^9^/L） | **Sham-operated group** | **2.17±0.49** | **1.74±0.35** | **2.00±1.14** | **2.81±0.66** | **3.41±1.41** | 0.83～4.91 |
|  | **Ccontrol group** | **2.55±0.31** | **1.88±0.30** | **2.20±0.61** | **3.31±1.53** | **2.07±0.31** |  |
|  | **Gel-nHAP group** | **2.30±0.25** | **1.59±0.55** | **2.43±0.34** | **2.41±0.23** | **2.47±0.48** |  |
|  | **cBMSCs-Gel-nHAP group** | **3.39±0.51** | **2.53±0.63** | **2.24±0.20** | **3.42±1.15** | **2.85±0.15** |  |
| MONO（10^9^/L） | **Sham-operated group** | **0.62±0.06** | **1.57±0.34** | **1.44±0.24** | **1.55±0.13** | **0.85±0.25** | 0.1～1.97 |
|  | **Ccontrol group** | **0.56±0.04** | **1.16±0.33** | **0.95±0.14** | **0.98±0.09** | **0.66±0.07** |  |
|  | **Gel-nHAP group** | **0.53±0.05** | **1.37±0.30** | **1.30±0.39** | **0.76±0.21** | **0.65±0.11** |  |
|  | **cBMSCs-Gel-nHAP group** | **0.39±0.09** | **0.90±0.03** | **1.46±0.62** | **1.00±0.12** | **0.59±0.02** |  |
| EOS（10^9^/L） | **Sham-operated group** | **0.43±0.11** | **0.28±0.18** | **0.33±0.18** | **0.43±0.06** | **0.50±0.17** | 0.04～1.62 |
|  | **Ccontrol group** | **0.28±0.28** | **0.07±0.05** | **0.29±0.15** | **0.28±0.12** | **0.21±0.08** |  |
|  | **Gel-nHAP group** | **0.42±0.42** | **0.2±0.23** | **0.28±0.22** | **0.27±0.05** | **0.23±0.11** |  |
|  | **cBMSCs-Gel-nHAP group** | **0.12±0.04** | **0.13±0.09** | **0.11±0.05** | **0.17±0.10** | **0.11±0.00** |  |
| BASO（%） | **Sham-operated group** | **0.05±0.05** | **0.03±0.01** | **0.03±0.02** | **0.04±0.01** | **0.05±0.02** | 0～0.12 |
|  | **Ccontrol group** | **0.02±0.01** | **0.01±0.00** | **0.02±0.01** | **0.03±0.04** | **0.03±0.03** |  |
|  | **Gel-nHAP group** | **0.02±0.02** | **0.02±0.00** | **0.01±0.01** | **0.01±0.00** | **0.01±0.00** |  |
|  | **cBMSCs-Gel-nHAP group** | **0.01±0.00** | **0.01±0.00** | **0.01±0.00** | **0.01±0.00** | **0.01±0.00** |  |
| RBC（10^12^/L） | **Sham-operated group** | **7.44±0.37** | **6.23±0.71** | **6.94±0.81** | **6.83±0.65** | **6.67±0.86** | 4.29～8.5 |
|  | **Ccontrol group** | **7.00±0.56** | **6.04±0.41** | **5.81±0.40** | **6.30±0.39** | **6.34±0.55** |  |
|  | **Gel-nHAP group** | **6.23±0.31** | **6.00±0.74** | **5.81±0.23** | **6.30±0.32** | **6.13±0.45** |  |
|  | **cBMSCs-Gel-nHAP group** | **7.17±0.81** | **6.72±0.59** | **6.71±0.57** | **5.59±0.26** | **5.89±0.72** |  |
| HGB（g/L） | **Sham-operated group** | **177.00±12.77** | **152.00±16.09** | **170.00±20.95** | **165.33±13.50** | **160.00±2.00** | 110～190 |
|  | **Ccontrol group** | **165.00±8.52** | **140.00±14.53** | **139.60±10.69** | **148.67±11.93** | **149.00±14.53** |  |
|  | **Gel-nHAP group** | **147.67±10.69** | **142.33±22.50** | **136.00±9.54** | **150.00±2.00** | **147.33±9.50** |  |
|  | **cBMSCs-Gel-nHAP group** | **169.00±19.47** | **160.00±15.72** | **159.00±13.96** | **132.67±9.02** | **140.67±17.62** |  |
| HCT（%） | **Sham-operated group** | **49.93±3.26** | **41.83±5.04** | **46.77±6.85** | **44.23±3.09** | **44.33±0.21** | 33～56 |
|  | **Ccontrol group** | **44.70±5.10** | **38.83±4.06** | **37.77±3.16** | **40.43±3.61** | **40.70±5.05** |  |
|  | **Gel-nHAP group** | **40.60±3.05** | **39.63±5.93** | **36.70±2.87** | **41.67±1.32** | **40.20±3.20** |  |
|  | **cBMSCs-Gel-nHAP group** | **47.13±5.39** | **44.10±4.07** | **44.20±3.73** | **35.53±3.65** | **38.93±4.72** |  |
| PLT（10^9^/L） | **Sham-operated group** | **282.67±67.53** | **182.00±16.86** | **258.67±59.72** | **262.33±65.68** | **302.33±84.97** | 117～490 |
|  | **Ccontrol group** | **381.67±80.41** | **336.67±84.51** | **291.00±33.05** | **388.00±180.03** | **485.33±161.97** |  |
|  | **Gel-nHAP group** | **310.00±71.19** | **283.67±29.02** | **322.67±01.79** | **354.33±20.55** | **340.00±48.66** |  |
|  | **cBMSCs-Gel-nHAP group** | **296.67±39.27** | **294.67±17.62** | **286.00±49.51** | **264.00±46.29** | **270.67±61.10** |  |

Table S3 Blood biochemical results（mean±SEM）

| **Indicators**  **（Unit）** | **Group** | **Before modeling** | 1D | 3D | 7D | **Normal range** |
| --- | --- | --- | --- | --- | --- | --- |
| TP（g/L） | **Sham-operated group** | **62.41±2.37** | **59.22±1.31** | **64.64±11.09** | **60.00±2.31** | 50~72 |
|  | **Ccontrol group** | **52.74±42.19** | **58.94±7.75** | **60.95±0.90** | **60.17±5.51** |  |
|  | **Gel-nHAP group** | **57.94±3.14** | **61.15±2.94** | **62.28±2.16** | **61.98±1.66** |  |
|  | **cBMSCs-Gel-nHAP group** | **64.42±10.78** | **62.75±7.41** | **64.79±7.15** | **63.14±4.16** |  |
| ALB（g/L） | **Sham-operated group** | **29.83±3.61** | **28.90±4.45** | **29.37±6.61** | **24.77±1.24** | 26~40 |
|  | **Ccontrol group** | **25.80±5.39** | **26.57±1.99** | **25.20±0.95** | **26.93±2.66** |  |
|  | **Gel-nHAP group** | **25.80±1.54** | **26.80±1.57** | **25.33±1.70** | **26.60±0.98** |  |
|  | **cBMSCs-Gel-nHAP group** | **24.87±1.98** | **23.03±1.88** | **23.57±1.07** | **22.87±1.14** |  |
| GLOB（g/L） | **Sham-operated group** | **29.67±6.35** | **30.33±5.69** | **35.33±5.03** | **34.33±3.05** | 16~37 |
|  | **Ccontrol group** | **33.33±9.02** | **32.67±9.45** | **36.00±0.00** | **33.00±7.94** |  |
|  | **Gel-nHAP group** | **32.33±3.21** | **34.67±2.52** | **37.00±1.00** | **36.67±1.53** |  |
|  | **cBMSCs-Gel-nHAP group** | **41.67±6.23** | **39.67±6.66** | **41.33±6.11** | **40.33±4.16** |  |
| TBIL（μmol/L） | **Sham-operated group** | **6.11±4.75** | **6.14±3.05** | **5.22±1.06** | **2.82±2.60** | 2~9 |
|  | **Ccontrol group** | **3.15±1.75** | **2.94±1.85** | **2.69±1.47** | **2.30±2.34** |  |
|  | **Gel-nHAP group** | **1.54±0.54** | **3.25±0.81** | **1.58±0.83** | **2.74±2.75** |  |
|  | **cBMSCs-Gel-nHAP group** | **2.28±0.29** | **1.76±0.07** | **2.14±0.80** | **1.55±0.62** |  |
| CK（U/L） | **Sham-operated group** | **141.37±15.39** | **1124.40±58.98** | **280.13±34.62** | **129.23±2511** | 24～195 |
|  | **Ccontrol group** | **131.03±29.17** | **1329.37±171.05** | **583.63±596.33** | **83.33±2.90** |  |
|  | **Gel-nHAP group** | **80.80±7.14** | **1347.63±635.89** | **326.23±90.45** | **102.23±47.17** |  |
|  | **cBMSCs-Gel-nHAP group** | **84.30±47.42** | **1470.37±1692.97** | **467.67±408.00** | **76.83±18.24** |  |
| ALP（U/L） | **Sham-operated group** | **32.6±11.77** | **84.77±41.95** | **78.23±15.20** | **60.33±9.02** | 20~110 |
|  | **Ccontrol group** | **57.47±26.63** | **105.83±63.83** | **63.57±15.81** | **72.90±46.93** |  |
|  | **Gel-nHAP group** | **49.20±9.33** | **97.33±16.59** | **107.90±29.40** | **73.43±20.23** |  |
|  | **cBMSCs-Gel-nHAP group** | **119.57±54.60** | **197.67±82.68** | **180.03±67.40** | **113.00±33.83** |  |
| ALT（U/L） | **Sham-operated group** | **100.90±100.93** | **67.17±45.34** | **69.77±52.55** | **45.53±12.84** | 17~78 |
|  | **Ccontrol group** | **69.53±36.13** | **60.27±13.28** | **55.27±15.12** | **51.03±7.62** |  |
|  | **Gel-nHAP group** | **31.03±1.91** | **41.60±5.28** | **41.93±4.74** | **40.30±6.35** |  |
|  | **cBMSCs-Gel-nHAP group** | **36.00±23.60** | **111.03±137.61** | **100.43±112.62** | **60.97±48.25** |  |
| GGT（U/L） | **Sham-operated group** | **10.13±0.32** | **10.67±0.61** | **10.60±0.53** | **6.97±4.70** | 5~14 |
|  | **Ccontrol group** | **7.70±2.21** | **7.70±2.07** | **5.37±1.80** | **6.33±3.17** |  |
|  | **Gel-nHAP group** | **5.97±1.61** | **6.03±2.05** | **4.90±1.54** | **7.37±4.66** |  |
|  | **cBMSCs-Gel-nHAP group** | **7.23±1.62** | **6.57±1.55** | **8.53±2.35** | **60.97±48.25** |  |
| BUN（mmol/L） | **Sham-operated group** | **4.64±2.66** | **5.68±0.73** | **5.30±1.10** | **4.84±1.40** | 2.5~9.6 |
|  | **Ccontrol group** | **4.87±1.73** | **3.62±1.34** | **3.46±0.93** | **4.65±1.08** |  |
|  | **Gel-nHAP group** | **4.34±0.45** | **2.97±0.82** | **2.94±0.70** | **4.72±1.04** |  |
|  | **cBMSCs-Gel-nHAP group** | **5.89±1.04** | **5.30±1.05** | **4.87±0.90** | **3.73±0.91** |  |
| CRE（μmol/L） | **Sham-operated group** | **54.23±9.87** | **75.37±50.19** | **50.43±7.45** | **63.20±11.90** | 35~124 |
|  | **Ccontrol group** | **52.33±11.26** | **44.80±6.79** | **55.57±12.71** | **53.33±9.21** |  |
|  | **Gel-nHAP group** | **66.43±9.12** | **54.77±5.00** | **59.17±14.16** | **58.13±9.05** |  |
|  | **cBMSCs-Gel-nHAP group** | **64.77±9.43** | **48.87±3.98** | **59.10±12.55** | **80.30±50.46** |  |
| Ca（mmol/L） | **Sham-operated group** | **2.38±0.11** | **1.48±1.27** | **2.25±0.41** | **2.14±0.21** | 2.33~3.03 |
|  | **Ccontrol group** | **2.56±0.21** | **2.30±0.13** | **2.41±0.26** | **2.46±0.24** |  |
|  | **Gel-nHAP group** | **2.67±0.12** | **2.27±0.38** | **2.51±0.01** | **2.48±0.50** |  |
|  | **cBMSCs-Gel-nHAP group** | **2.52±0.06** | **2.49±0.02** | **2.53±0.07** | **2.47±0.12** |  |
| AST（mmol/L） | **Sham-operated group** | **22.90±2.65** | **51.63±5.75** | **19.23±1.23** | **19.73±2.65** | 0～40 |
|  | **Ccontrol group** | **28.03±2.29** | **75.37±0.93** | **82.90±97.70** | **20.37±2.51** |  |
|  | **Gel-nHAP group** | **18.70±0.70** | **74.67±30.37** | **32.33±4.44** | **19.07±2.12** |  |
|  | **cBMSCs-Gel-nHAP group** | **26.27±13.50** | **207.47±301.59** | **50.00±32.20** | **21.63±4.62** |  |
